# Anti-Osteoarthritic Effects of the *Litsea japonica* Fruit in a Rat Model of Osteoarthritis Induced by Monosodium Iodoacetate

**DOI:** 10.1371/journal.pone.0134856

**Published:** 2015-08-05

**Authors:** Yong Joon Jeong, Inhye Kim, Joon Hyung Cho, Dae Won Park, Jung Eun Kwon, Moon Won Jung, Xue Meng, Se Min Jo, Hae Seong Song, Young Mi Cho, Sang Mok Song, Young-Min Ham, Yong-Hwan Jung, Chang Sook Kim, Weon-Jong Yoon, Se Chan Kang

**Affiliations:** 1 Department of Life Science, Gachon University, Seongnam, Republic of Korea; 2 Department of Food and Nutrition, Hanyang University, Seoul, Republic of Korea; 3 Department of Biological & Environmental Science, Dongguk University, Seoul, Republic of Korea; 4 Jeju Biodiversity Research Institute, Jeju Technopark, Jeju, Republic of Korea; National Center for Scientific Research Demokritos, GREECE

## Abstract

Osteoarthritis (OA) is a degenerative chronic disease that affects various tissues surrounding the joints, such as the subchondral bone and articular cartilage. The onset of OA is associated with uncontrolled catabolic and anabolic remodeling processes of the joints, including the cartilage and subchondral bone, to adapt to local biological and biochemical signals. In this study, we determined whether 70% ethanolic (EtOH) extract of *Litsea japonica* fruit (LJFE) had beneficial effects on the articular cartilage, including structural changes in the tibial subchondral bone, matrix degradation, and inflammatory responses, in OA by using a rat model of monosodium iodoacetate-induced OA. Our results showed that administration of LJFE increased the bone volume and cross-section thickness, but the mean number of objects per slice in this group was lower than that in the OA control (OAC) group. In addition, the LJFE decreased the expression of inflammatory cytokines. Compared to the OAC group, the group treated with high doses of LJFE (100 and 200 mg/kg) showed a more than 80% inhibition of the expression of matrix metalloproteinases and tissue inhibitors of metalloproteinases. Our results suggest that LJFE can be used as a potential anti-osteoarthritic agent.

## Introduction

Osteoarthritis (OA) is a degenerative disease and is a major cause of disability and pain, especially in the elderly population [1]. OA is caused by many factors, and although aging is the most common factor associated with the development of OA, other factors such as hormonal, mechanical, and genetic factors also lead to the development of OA [2]. Further, OA is characterized by the progressive loss of articular cartilage and formation of osteophytes and is associated with degeneration of the cartilage and changes to the subchondral bone, which lead to chronic pain and functional limitations in the joint [3,4].

Degradation of the articular cartilage in OA is mediated by excessive synthesis and release of catabolic and inflammatory factors such as matrix metalloproteinases (MMPs), tissue inhibitor of metalloproteinases (TIMPs), and cytokines [5]. MMPs play an important role in tissue remodeling and in destruction of the cartilage and bone in arthritic joints [6]. TIMPs are important controllers of MMPs that participate in the degradation of the extracellular matrix [7,8]. In addition, cartilage degradation in OA is recognized to be induced by inflammatory cytokines such as interleukin-6 (IL-6), IL-1β, and tumor necrosis factor-α (TNF-α) [9]. Therefore, MMPs, TIMPs, and inflammatory cytokines are reasonable therapeutic approaches for the treatment of OA [8].

*Litsea japonica* Jussieu (Lauraceae) is an endemic plant, which grows in the southern areas of Korea and Japan. This plant is used as a food in Korea, and it contains different kinds of essential oils, fatty acids, lactones, alkaloids, and terpenoids [10–12]. Previous studies have reported that this plant has anti-inflammatory [13], anti-oxidant [14], anti-diabetic effects [15]. In addition, the components of the *L*. *japonica* fruit, hamabiwalactone A and B, which have anti-inflammatory and analgesic activities, were separated in previous studies [16]. However, the effects of *L*. *japonica* fruits on OA have not yet been investigated thus far. Therefore, in this study, we investigated the effects of 70% ethanolic (EtOH) extract of *L*. *japonica* fruit (LJFE) on the degradation of the articular cartilage, including structural changes in the tibial subchondral bone, matrix degradation, and inflammatory responses by using a monosodium iodoacetate (MIA)-induced rat model of OA.

## Materials and Methods

### Preparation of LJFE

*L*. *japonica* fruits used in this study were collected from Jeju Island in Korea through Jeju Biodiversity Research Institute supported by the Jeju Technopark. A voucher specimen (No. JBRI-079) was deposited in the Jeju Biodiversity Research Institute. The dried fruit (1,000 g) was extracted twice with 70% EtOH for 18 h each at 60°C, and the extract was concentrated under reduced pressure. The decoction was filtered and lyophilized to yield the LJFE. The yield of dried extract from crude material was approximately 49.6% (w/w) [16], and it was stored at 4°C until use.

### HPLC analysis of the LJFE

To measure the content of active ingredients, we analyzed the extract to determine the presence of hamabiwalactone A and B. Chromatographic analysis of the LJFE was performed using an HPLC with an Alliance Waters 2695 separation module coupled to a Waters 2998 photodiode array detector, utilizing a cadenza CD-18 4.6 mm × 150 mm C18 column (particle size, 5 μ; Imtakt, USA) at a flow rate of 1.2 mL/min. The column was placed in a column oven at 25°C. The ratio of the mobile phases A (0.5% acetic acid) and B (acetonitrile) were changed after 0 (6:4, v/v), 30 (0:10, v/v), and 40 (6:4, v/v) min, the injection volume was 5 μl, and UV detection was performed at 254 nm. The quantification of hamabiwalactone A and B in the LJFE was performed using the external standard method, and pure hamabiwalactone A and B were used as the standard stock solutions (12.5, 25, 50, 100, and 200 μg/mL) [16].

### Experimental animals

We obtained 5-week-old male Sprague—Dawley rats (200–210 g) from RaonBio Inc. (Yongin, Korea) for our studies. The animals were acclimated for 1 week and housed under controlled conditions of temperature (23 ± 2°C) and humidity (55±7%) in a 12-h light/12-h dark cycle and ventilation of 10–15 times/h (on an all-fresh-air basis), and they had free access to sterile food and water. The experimental animal facility and protocols were approved by the Institutional Animal Care and Use Committee of Gachon University (GIACUC-R2013017B). All experimental procedures were performed in compliance with the NIH Guide for the Care and Use of Laboratory Animals and National Animal Welfare Law in Korea.

### Experimental model of MIA-induced OA in rats

The animals were randomized and assigned to treatment groups before the initiation of the study (n = 6 per group). The rats were maintained under anesthesia via inhalation with isoflurane (Hana Pharm, Korea) and were given a single intra-articular injection of MIA (Sigma, USA) through the infrapatellar ligament of the right knee [17]. MIA was dissolved to a final concentration of 60 mg/mL in 0.9% sterile saline, and 50 μL of the solution was administered using a 26.5-G needle. Normal control (NC) rats were injected with an equivalent volume of saline. LJFE and indomethacin (Sigma, USA) were dissolved in saline and administered orally every day for 3 weeks after injection of MIA. The dose of LJFE was 50, 100, and 200 mg/kg, and that of indomethacin was 2 mg/kg. The body weight was measured once a week.

### Micro-computed tomography analysis

To evaluate the structural changes in the bone architecture, the proximal and distal part of the right tibiae were scanned using *in vivo* micro-computed tomography (Micro-CT, Skyscan, Belgium). The scan conditions were as follows: an aluminum filter of 0.5 mm, X-ray voltage of 50 Kv, X-ray current of 200 mA, and an exposure time of 360 ms. During each scan, the rats were anesthetized with ketamine (1.5 mL/kg, Huons, Korea) and xylazine (0.5 mL/kg, Bayer, Germany) by using an intraperitoneal injection. Three-dimensional models of the tibiae were reconstructed using SkyScan CT Analyzer version 1.11. In addition, the structural parameters were measured at the subchondral bone for the knee and the tibiae.

### Serum analysis

After the rats were killed, the samples of whole blood were collected from the abdominal vein. Blood was allowed to clot for 30 min. Then, the serum was separated via centrifugation at 1,500 *g* for 10 min. The levels of cytokines in the serum were measured using enzyme-linked immunosorbent assay (ELISA) kits for IL-6, IL-1β, and TNF-α (R&D Systems, USA). All ELISA procedures were performed according to the manufacturers’ protocols.

### Quantitative real-time polymerase chain reaction analysis

The sample for extracting the RNA was obtained from the cartilage tissue of rats using cryogenic grinding, and total RNA was extracted by using the PureLink RNA Mini Kit (Ambion, USA) according to the manufacturer’s instructions. We transcribed 1 μg of total RNA in a 20-μL volume by using oligo (dT) primers, with the enzyme and buffer supplied in the PrimeScript II 1^st^ strand cDNA Synthesis kit (Takara, Japan). Quantitative real-time polymerase chain reactions (PCR) were performed using a MX3005P (Stratagene, USA) by using the following primers (Table 1). For real-time PCR, SYBR Premix Ex Taq II (Takara, Japan) was used. The final volume of the reaction mixture was 25 μL containing 2 μL cDNA template, 12.5 μL master mix, 1 μL each primer (10 μM stock solution), and 8.5 μL sterile distilled water. The thermal cycling profile consisted of a pre-incubation step at 95°C for 10 min, followed by 40 cycles at 95°C (15 s) and 60°C (60 s). Relative quantitative evaluation of MMP-2, MMP-3, MMP-7, MMP-9, MMP-13, TIMP-1, and TIMP-2 was performed using comparative CT (cycle threshold) [18].

**Table 1:** The primer sequences used for real-time polymerase chain reaction (PCR).

Gene name^a^	Primer sequences
GAPDH	5′-GCTTAAGAGACAGCCGCATCT-3′ (sense)
	5′-CGACCTTCACCATTTTGTCTACA-3′ (antisense)
MMP-2	5′-TCCCGAGATCTGCAAGCAAG-3′ (sense)
	5′-AGAATGTGGCCACCAGCAAG-3′ (antisense)
MMP-3	5′-TGATGGGCCTGGAATGGTC-3′ (sense)
	5′-TTCATGAGCAGCAACCAGGAATAG-3′ (antisense)
MMP-7	5′-GACATTGCAGGCATCCAGAAGTTA-3′ (sense)
	5′-AGGGCGTTTGCTCATTCCAG-3′ (antisense)
MMP-9	5′-AGCCGGGAACGTATCTGGA-3′ (sense)
	5′-TGGAAACTCACACGCCAGAAG-3′ (antisense)
MMP-13	5′-CCCTGGAATTGGCGACAAAG-3′ (sense)
	5′-GCATGACTCTCACAATGCGATTAC-3′ (antisense)
TIMP-1	5′-CATCTCTGGCCTCTGGCATC-3′ (sense)
	5′-CATAACGCTGGTATAAGGTGGTCTC-3′ (antisense)
TIMP-2	5′-GACACGCTTAGCATCACCCAGA-3′ (sense)
	5′-CTGTGACCCAGTCCATCCAGAG-3′ (antisense)

^a^GAPDH, glyceraldehyde 3-phosphate dehydrogenase; MMPs, matrix metalloproteinases; TIMPs, tissue inhibitor of metalloproteinases

### Statistical analysis

Data is represented as mean ± standard error of the mean (SEM). Group differences were determined using one-way analysis of variance (ANOVA) with Dunnett’s test, followed by a modified *t*-test with Bonferroni correction for comparisons between individual groups. Significant values are represented by an asterisk (*p < 0.05 or **p < 0.01 compared to NC, ^#^p < 0.05 or ^##^p < 0.01 compared to osteoarthritis control group [OAC]).

## Results and Discussion

### Analysis of the content of active ingredients in LJFE

**Fig 1:**
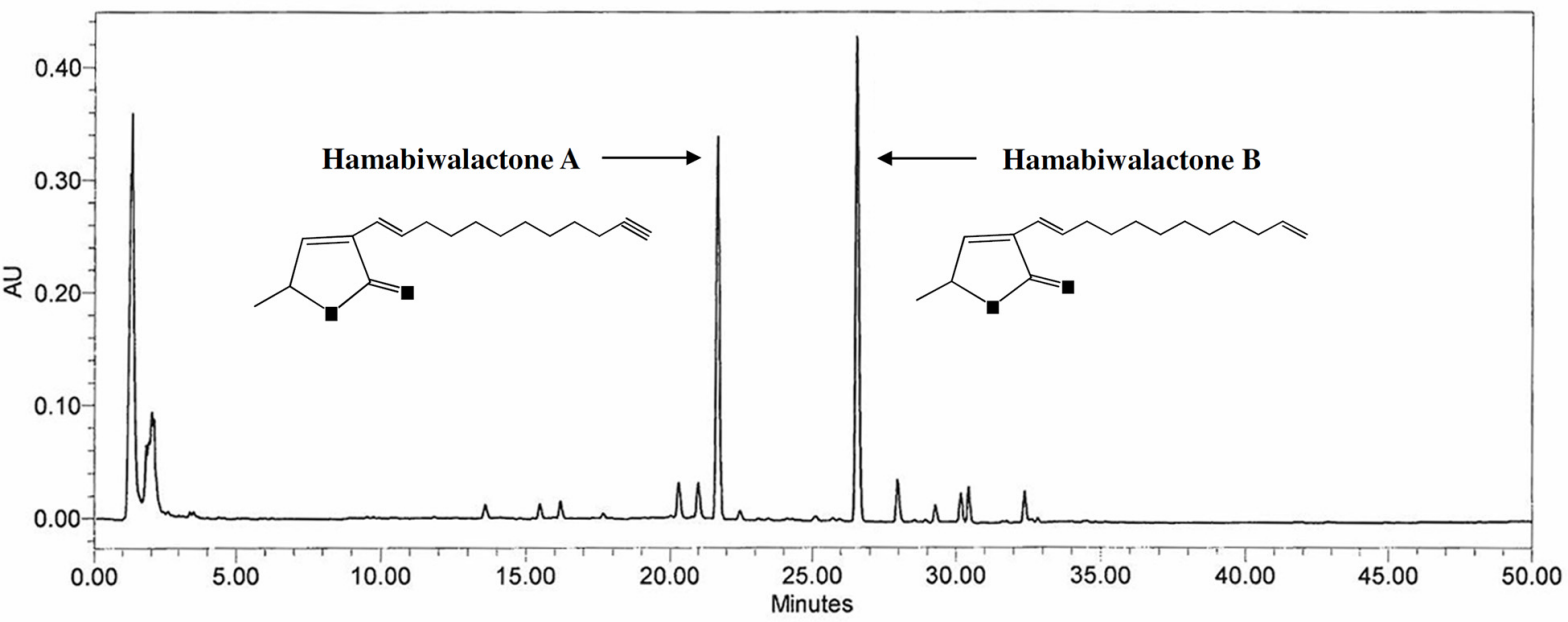
HPLC chromatogram of the 70% ethanolic (EtOH) extract of *Litsea japonica* fruit (LJFE). HPLC was performed using a Symmetry C18 column, 5 μL injection at 30°C, and detection at 254 nm.

In previous studies, we separated the active ingredients of LJFE hamabiwalactone A (3-[(1E)-1-dodecen-11-yn-1yl]-5-methyl-2(5H)-furanone, C_17_H_26_O_2_) and hamabiwalactone B (2(5H)-furanone,3-(1E)-1,11-dodecadien-1-yl-5-methyl-, (5S)-, C_17_H_26_O_2_), which have anti-inflammatory and analgesic effects [16]. OA is characterized by joint pain, and inflammation is substantially involved in the pathogenesis and progression of OA [1,8]. Therefore, hamabiwalactone A and B present in the *L*. *japonica* fruit may be used as active ingredients for the treatment of OA, and in this study, we measured the levels of hamabiwalactone A and B in the LJFE. Our results showed that content of hamabiwalactone A in the LFJE was 12.1 ± 0.08 mg/g and that of hamabiwalactone B was 15.9 ± 0.09 mg/g (Fig 1).

### Development of an MIA-induced rat model of OA and weight gain

MIA is an inhibitor of glyceraldehyde-3-phosphate dehydrogenase and it disrupts chondrocyte glycolysis [19]. An intra-articular injection of MIA induces chondrocyte death in the articular cartilage and leads to symptoms of OA [17]. Therefore, the injection of MIA into the intra-articular space of the rat knee is used widely as a chemically induced model of OA in preclinical efficacy studies [20, 21]. In this study, MIA-induced OA rats were administered LJFE (50, 100, and 200 mg/kg) for 3 weeks, and then, the body weight, structural properties of the subchondral bone, and the levels of OA biomarkers in the serum and cartilage were measured to determine the therapeutic effects of LJFE in OA.

The final body weight and body weight gain of the animals in each group are shown in Table 2. No significant differences were observed in body weight gain between the NC, MIA-induced OA control (OAC), or OA treated with LJFE groups, while OA treated with indomethacin administered group (IM, 109.2 ± 12.51 g) showed a significantly (p < 0.05) lower weight gain than the OAC group (120.4 ± 6.78 g).

**Table 2:** Body weight and weight gain in rats with osteoarthritis.

	Body weight (g)		
Group^a^	Initial	Final	Weight gain (g)
NC	225.1 ± 8.07	347.6 ± 10.55	122.4 ± 8.99
OAC	206.3 ± 11.91**	326.8 ± 10.17**	120.4 ± 6.78
LJFE 50	213.0 ± 7.71	326.9 ± 15.51	113.9 ± 12.48
LJFE 100	207.9 ± 10.75	327.7 ± 9.21	119.8 ± 8.03
LJFE 200	210.3 ± 8.72	329.8 ± 16.76	119.4 ± 17.18
IM	207.1 ± 9.01	316.3 ± 13.13	109.2 ± 12.51^#^

^a^NC, normal control; OAC, OA control; LJFE 50, OA+LJFE 50 mg/kg; LJFE 100, OA+LJFE 100 mg/kg; LJFE 200, OA+LJFE 200 mg/kg; IM, indomethacin 2 mg/kg. Values represent the mean ± S.D. (n = 9).

** p < 0.01 compared to NC,

^#^p < 0.05 compared to OAC.

### Effect of LJFE on the structural properties of the subchondral bone

Recent studies have used micro-CT to quantify the structural changes in the tibial subchondral bone in a MIA-induced OA rat model [21]. To investigate the effect of LJFE on structural characteristics, the tibia of each rat was scanned after the experimental and structural parameters for the entire lateral and medial subchondral bone of the tibiae, bone volume density (BV, mm^3^), mean number of objects per slice (Obj.N.), and cross-section thickness (Cs.Th, mm) were measured and calculated by using 3D micro-CT images.

**Fig 2:**
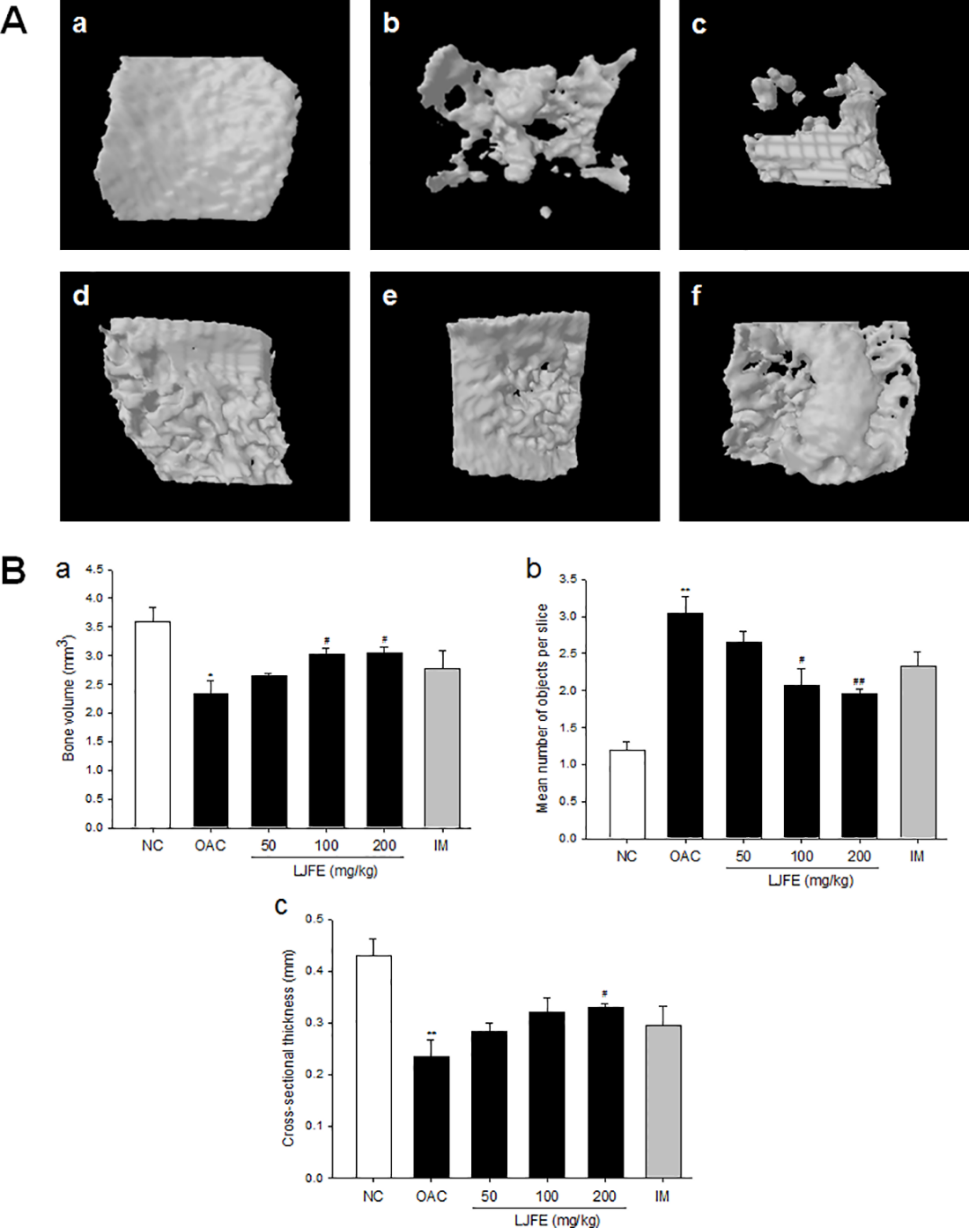
Three-dimensional micro-computed tomography images and parameters of lateral subchondral bone of the knee of the tibiae. (A) 3D micro-computed tomography (CT) images of the lateral subchondral bone. (a) Normal control, (b) OA control, (c) OA+70% ethanolic extract of *Litsea japonica* fruit (LJFE) 50 mg/kg, (d) OA+LJFE 100 mg/kg, (e) OA+LJFE 200 mg/kg, and (f) indomethacin 2 mg/kg. (B) The lateral subchondral bone parameters were monitored using a 3D micro-CT analysis program. The lateral subchondral bone parameters assessed were as follows: (a) bone volume (BV, mm^3^), (b) mean number of objects per slice (Obj.N.), and (c) cross-sectional thickness (Cs.Th, mm). The results are mean ± standard error of mean (SEM) for six rats per group (*p < 0.05, **p < 0.01 compared to NC, ^#^p < 0.05, ^##^p < 0.01 compared to OAC).

OA rats treated with LJFE and IM showed a lesser loss of the lateral subchondral bone than rats in the OAC group (Fig 2A). In addition the BV and Cs.Th were significantly higher, whereas the Obj.N. was significantly lower in the groups treated with LJFE and IM than in the OAC group (Fig 2B). In particular, the group receiving 200 mg/kg of LJFE for 3 weeks showed a significantly greater increase in the BV (3.1 ± 0.10 mm^3^, p < 0.05) and Cs.Th (0.3 ± 0.01 mm, p < 0.05) than the OAC group (2.3 ± 0.22 mm^3^ and 0.2 ± 0.03 mm, respectively); however, the Obj.N. (2.0 ± 0.06, p < 0.01) in this group was lower than that in the OAC group (3.0 ± 0.23) (Fig 2B).

**Fig 3:**
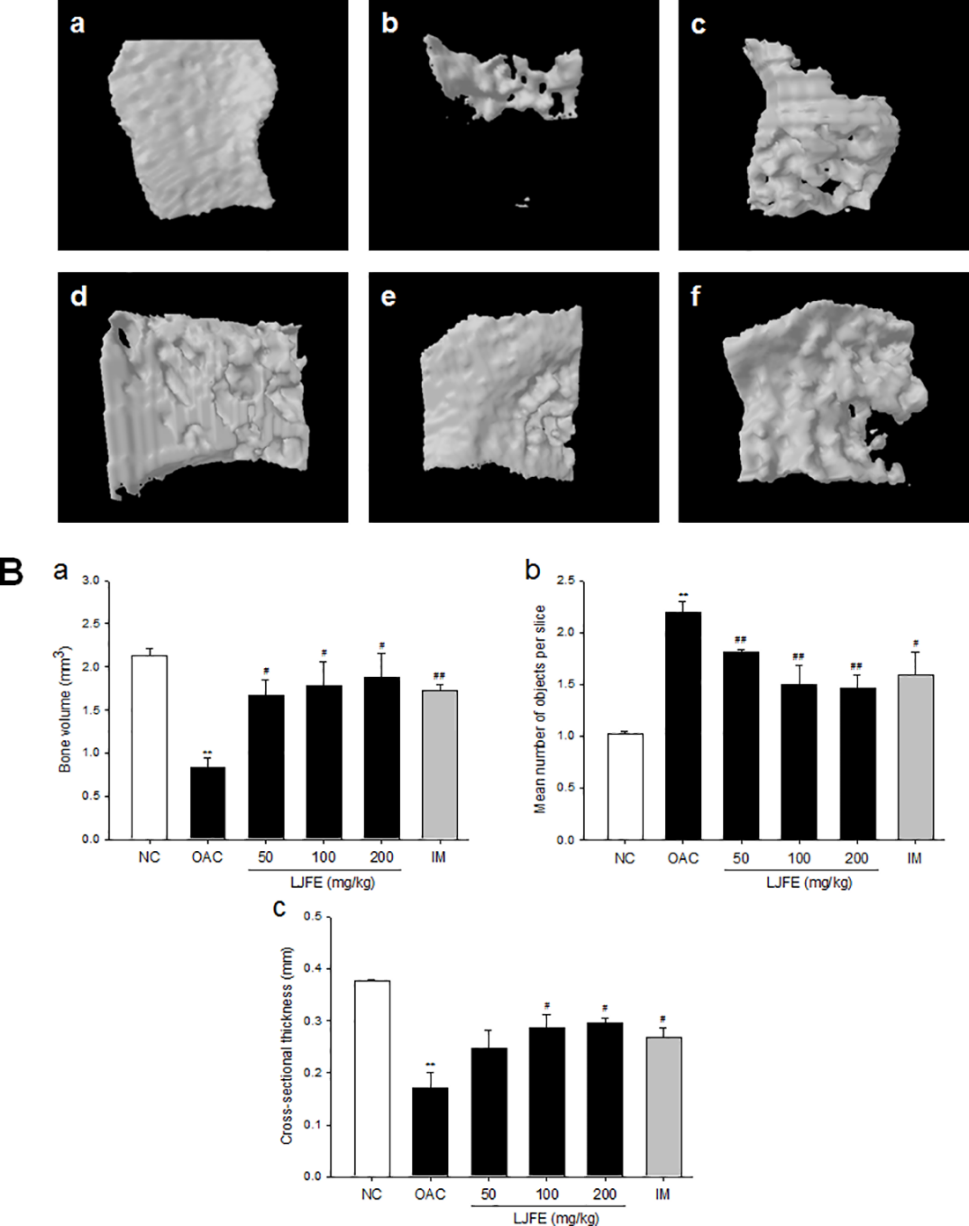
Three-dimensional micro-computed tomography (CT) images and parameters of medial subchondral bone for the knee of the tibiae. (A) 3D micro-computed tomography (CT) images of the medial subchondral bone. (a) Normal control, (b) OA control, (c) OA+70% ethanolic extract of *Litsea japonica* fruit (LJFE) 50 mg/kg, (d) OA+LJFE 100 mg/kg, (e) OA+LJFE 200 mg/kg, and (f) indomethacin 2 mg/kg. (B) The medial subchondral bone parameters were monitored using a 3D micro-CT analysis program. The medial subchondral bone parameters assessed were as follows: (a) bone volume (BV, mm^3^), (b) mean number of objects per slice (Obj.N.), and (c) cross-sectional thickness (Cs.Th, mm). The results are mean ± standard error of mean (SEM) for six rats per group (**p < 0.01 compared to NC, ^#^p < 0.05, ^##^p < 0.01 compared to OAC).

The medial subchondral bone showed structural characteristics similar to the lateral subchondral bone (Fig 3A). The BV and Cs.Th were significantly higher and the Obj.N. was significantly lower in the groups treated with LJFE and IM than in the OAC group (Fig 3B). Compared to the OAC group (0.8 ± 0.10 mm^3^ and 0.2 ± 0.03 mm, respectively), the group treated with 200 mg/kg of LJFE showed a marked increase in BV (1.9 ± 0.27 mm^3^, p < 0.05) and Cs.Th (0.3 ± 0.01 mm, p < 0.05), whereas the Obj.N. in this group (1.5 ± 0.13, p < 0.01) was lower than that in the OAC group (2.2 ± 0.11) (Fig 3B). Thus, treatment with LJFE (50, 100, and 200 mg/kg) showed significant and dose-dependent effects on MIA-induced architectural deterioration of the subchondral bone.

### Effect of LJFE on the serum levels of inflammatory cytokines

Inflammation is an important factor associated with the development and progression of OA [22, 23]. IL-1β and TNF-α are the most prominent inflammatory cytokines in OA. In vitro studies have showed that these cytokines are expressed and they stimulate the production of other cytokines such as IL-8 and IL-6 [24]. In a previous study, we investigated the inhibitory effects of LJFE on inflammatory cytokines using Raw 264.7 macrophage cells [16]. Therefore, in this study, we determined whether LJFE had any effects on the inflammatory cytokines associated with OA, such as IL-6, IL-1β, and TNF-α, by using MIA-induced OA rats.

**Fig 4:**
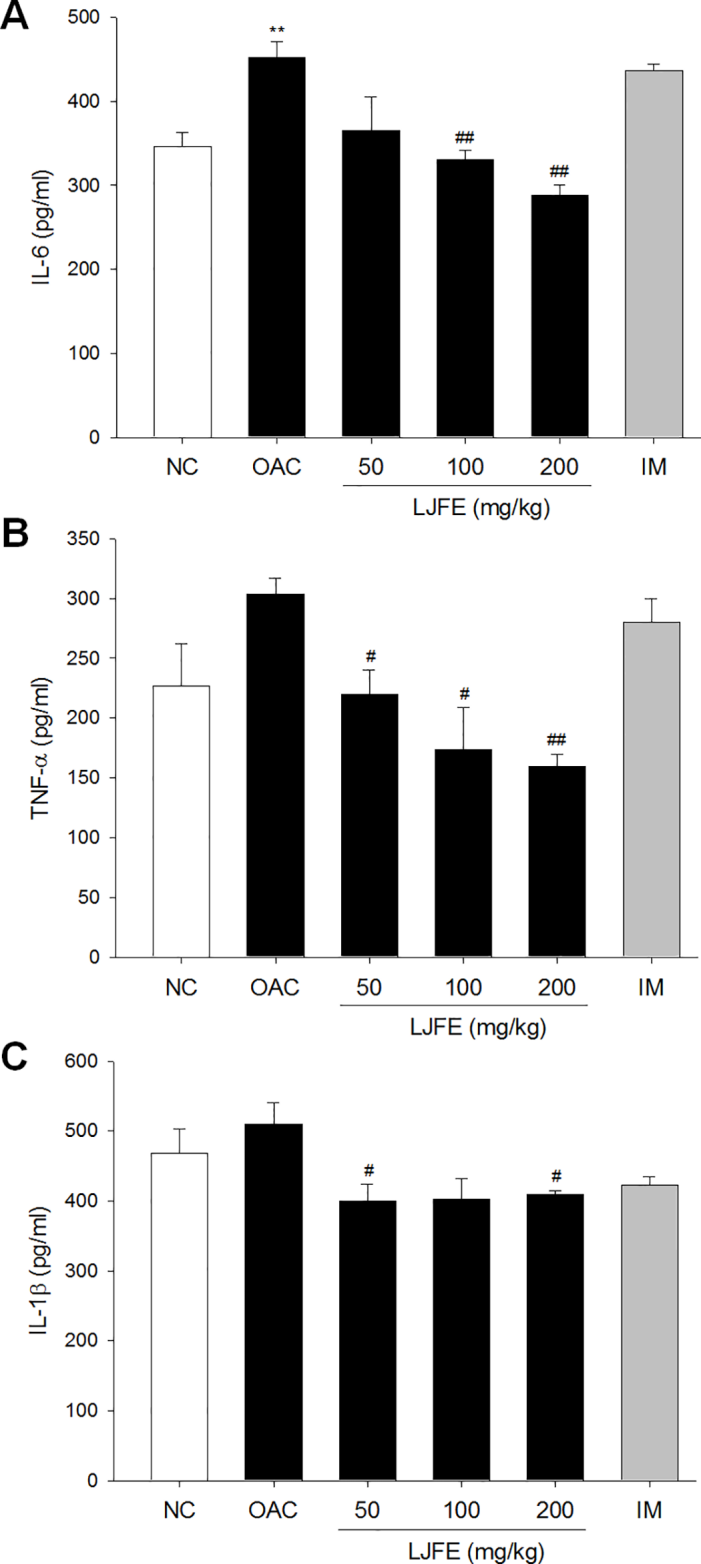
Levels of interleukin 6 (IL-6) (A), tumor necrosis factor-α (TNF-α) (B), and IL-1β (C) in the serum of rats with osteoarthritis. The levels of cytokines in the serum were determined using an enzyme-linked immunosorbent assay (ELISA). The results are mean ± standard error mean (SEM) for six rats per group (**p < 0.01 compared to NC, ^#^p < 0.05, ^##^p < 0.01 compared to OAC).

The serum levels of IL-6, IL-1β, and TNF-α were higher in the OAC group than in the NC group, and LJFE showed a significant and dose-dependent decrease in these levels, except those of IL-1β, compared to those in the OAC group (Fig 4A and 4B). Compared to the LJFE group, the OAC group did not show a dose-dependent decrease in IL-1β (Fig 4C). In particular, the group treated with 200 mg/kg of LJFE effectively inhibited the production of IL-6 (288.3 ± 11.67 pg/mL, p < 0.01), TNF-α (160.0 ± 10.00 pg/mL, p < 0.01), and IL-1β (410.0 ± 5.00 pg/mL, p < 0.05) compared to that in the OAC group (451.7 ± 18.78, 303.3 ± 13.33, and 510.0 ± 31.22 pg/mL, respectively).

### Effect of LJFE on the expression of MMPs and TIMPs

The progressive destruction of articular cartilage is caused by a number of matrix-degrading enzymes produced by the chondrocytes and synovium [25]. Among the various biomarkers associated with OA, MMPs play a primary role in the downstream signaling pathways in OA and cartilage degradation [26, 27]. MMPs are regulated by TIMP-1 and TIMP-2, and overexpression of MMPs results in an imbalance between the activity of MMPs and TIMPs that can cause a variety of pathological disorders [28–30]. To determine whether LJFE may affect the destruction of articular cartilage, we examined the expression of the OA biomarkers MMP-2, MMP-3, MMP-7, MMP-9, MMP-13, TIMP-1, and TIMP-2 in the articular cartilage of MIA-induced OA rats.

**Fig 5:**
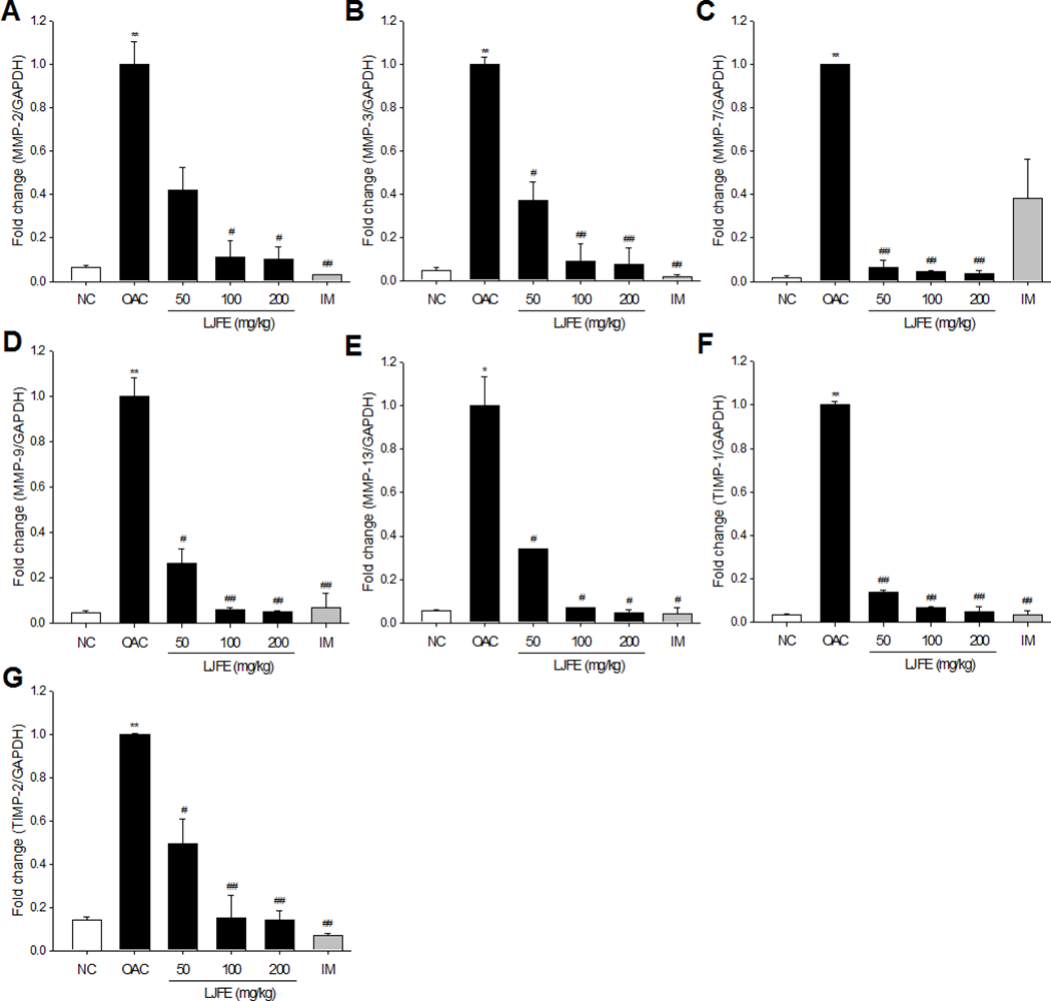
Levels of matrix metalloproteinase 2 (MMP-2) (A), MMP-3 (B), MMP-7 (C), MMP-9 (D), MMP-13 (E), tissue inhibitor of metalloproteinase 1 (TIMP-1) (F), and TIMP-2 (G) in the cartilage tissue. RNA was extracted from the cartilage tissue of rats with osteoarthritis, and the mRNA levels of MMPs and TIMPs were determined using real-time polymerase chain reaction (PCR). Glyceraldehyde-3-phosphate dehydrogenase (GAPDH) served as a house-keeping gene. The results are mean ± standard error of mean (SEM) for six rats per group (*p < 0.05, **p < 0.01 compared to NC, ^#^p < 0.05, ^##^p < 0.01 compared to OAC).

The OAC group showed a markedly higher mRNA expression of MMP-2, MMP-3, MMP-7, MMP-9, MMP-13, TIMP-1, and TIMP-2 in the articular cartilage (Fig 5). However, compared to OAC group, the group treated with LJFE and IM showed a decrease in the mRNA expression of MMP-2, MMP-3, MMP-7, MMP-9, MMP-13, TIMP-1, and TIMP-2 (Fig 5A–5G). High doses of LJFE (100 and 200 mg/kg) showed a significant inhibition of more than 80% of the expression of all these genes. These results indicated that LJFE directly modulated the expression of MMPs but not via TIMP stimulation. Possible explanations could be that LJFE have another active component with the MMP-like activity, thereby exerting the MMP-like activity. Extended experiments will be continued to figure out the mechanism by which LJFE inhibits the expression of MMPs.

## Conclusions

Previous studies showed that the *L*. *japonica* fruit had anti-inflammatory and analgesic effects. Because OA is associated with inflammation and pain, we examined the effects of LJFE in an MIA-induced OA model. Our results showed that LJFE inhibited the loss of lateral and medial subchondral bone of the knee of the tibiae. In addition, LJFE inhibited the expression of inflammatory markers such as IL-6, IL-1β, and TNF-α in the serum, which reduced the expression of biomarkers associated with OA, such as MMPs and TIMPs in the cartilage. Therefore, LJFE could be used as a potential candidate for the prevention and treatment of OA.
